# On the Adiabatic Shear Band Sensitivity of Extruded Ti-6Al-4V Alloy Under Dynamic Compression Along the Extrusion and Transverse Directions

**DOI:** 10.3390/ma19050955

**Published:** 2026-03-01

**Authors:** Chenxing Zheng, Weikang Fu, Tianyuan Gong, Yingqian Fu, Xinlu Yu

**Affiliations:** 1 Key Laboratory of Impact and Safety Engineering (Ningbo University), Ministry of Education, Ningbo 315211, China; xing220044@outlook.com (C.Z.); 2311090104@nbu.edu.cn (W.F.); gty15364898950@163.com (T.G.); 1111081012@nbu.edu.cn (Y.F.); 2 Faculty of Mechanical Engineering and Mechanics, Ningbo University, Ningbo 315211, China; 3 College of Science and Technology, Ningbo University, Ningbo 315300, China

**Keywords:** Ti-6Al-4V, texture, anisotropy, adiabatic shear band, ASB sensitivity, slip systems, CPFEM simulation

## Abstract

Adiabatic shear banding (ASB) is a critical failure mechanism in titanium alloys subjected to high-strain-rate deformation, and its initiation is strongly influenced by the initial crystallographic texture. The dynamic response and ASB sensitivity of extruded and annealed Ti-6Al-4V (TC4) alloy rods were investigated under dynamic compression of cubic specimens along the extrusion direction (ED) and the transverse direction (TD) at a strain rate of 2500 s^−1^. Split Hopkinson pressure bar (SHPB) tests combined with digital image correlation (DIC) were employed to obtain the stress–strain response and the evolution of strain localization. A dislocation density-based crystal plasticity finite element model (CPFEM), incorporating the measured texture, was established to elucidate the correlation between texture and ASB behavior. The experimental results show that TD specimens exhibit a yield strength approximately 100 MPa higher than that of ED specimens, while both orientations display comparable post-yield hardening behavior. ASB initiation occurs earlier in TD (compressive strain ~0.13) than in ED (~0.23), indicating greater ASB sensitivity in the TD orientation. The CPFEM successfully reproduces the directional stress–strain responses and the observed localization morphology, enabling mechanistic interpretation in terms of slip activity and thermomechanical coupling. The simulations indicate that ED loading is dominated by prismatic ⟨a⟩ slip, resulting in lower flow stress and more dispersed strain localization. In contrast, TD loading is governed primarily by pyramidal ⟨c + a⟩ slip, leading to elevated flow stress and intensified localization. The higher ASB sensitivity in the TD orientation is therefore attributed to texture-controlled slip-mode partitioning, enhanced thermomechanical coupling, and a more concentrated crystallographic orientation distribution that facilitates intergranular slip transfer. These findings provide guidance for tailoring microtexture to mitigate dynamic failure in titanium alloys subjected to high-strain-rate loading.

## 1. Introduction

Adiabatic shear bands (ASBs) are thermomechanical instabilities that arise under high-strain-rate deformation. Depending on the material, their width typically ranges from approximately 10 μm to several hundred micrometers. ASBs are widely observed in aerospace components, machining operations, and particularly in ballistic impact scenarios. In most cases, the formation of ASBs degrades material performance and leads to premature failure. However, in high-speed metal forming processes, the intentional induction and utilization of ASBs can enhance processing efficiency [1,2]. Consequently, ASBs have long been a subject of interest in engineering applications, especially in materials with inherently low thermal conductivity and specific heat capacity [3].

Titanium alloys, especially Ti-6Al-4V, are widely employed in the aerospace, automotive, and biomedical industries owing to their exceptional strength-to-weight ratio, corrosion resistance, and biocompatibility. Nevertheless, their low thermal conductivity makes them highly prone to ASB formation under dynamic loading conditions [4]. Ti-6Al-4V consists of a dual-phase α + β microstructure, where the α phase has a hexagonal close-packed (HCP) crystal structure. This crystallography often results in strong basal or prismatic textures after rolling, extrusion, or annealing. Achieving a uniform microstructure with random texture remains a significant challenge [5,6], contributing to the pronounced orientation dependence and significant anisotropy throughout the ASB evolution of titanium alloys.

Over the past few decades, numerous studies have investigated the role of microstructure and texture in ASB formation, with most focusing on post-deformation analysis. For example, Huang et al. [7] found that thermally compressed TC17 alloy exhibited texture rearrangement near the ASB, leading to prismatic slip dominating the deformation and promoting strain localization. Similarly, Chen et al. [8] reported that texture rearrangement within the ASB region of Ti-6Al-4V (TC4) alloy led to grain-scale heterogeneity. This soft–hard contrast accelerates microvoid nucleation, thereby promoting ASB formation and failure.

Despite these advances, the influence of the initial texture on the mechanical response and ASB formation in titanium alloys has received relatively limited attention. As early as 1986, McDarmaid and Partridge [9] identified texture as a key factor governing the deformation behavior and anisotropy of TC4 alloys. Coghe et al. [10] combined multi-strain-rate experiments with EBSD analysis and revealed that extruded TC4 exhibits strong transverse α-phase texture, with most *c*-axes oriented perpendicular to the extrusion direction (ED), which restricts the activation of slip systems with low critical resolved shear stress (CRSS) and promotes a more rapid transition from conventional slip to ASL. Similar anisotropic behavior was reported by Gu and Nesterenko [11]. More recently, Qin et al. [12] further demonstrated that rolling-induced texture in TC4 limits slip activity in the RD and TD directions, promoting strain localization under dynamic compression. Their results indicated that texture strengthening in the RD and TD directions limited slip activity, making them more susceptible to strain localization. Collectively, these studies suggest that deformation anisotropy and localization tendency in titanium alloys are closely linked to their initial texture and that specific textures can significantly promote ASB formation. However, existing studies have primarily focused on initial microstructural characteristics and post-mortem analysis of deformed specimens, while paying insufficient attention to the role of texture evolution during deformation in the development of localization. Furthermore, although it is well established that initial texture influences the activation of slip systems, the evolution of different slip modes under different texture conditions remains inadequately understood. Zhang et al. [13] addressed this gap by modeling ASB evolution using a dislocation-based crystal plasticity framework, revealing that favorable or unfavorable grain orientations can either promote or inhibit ASB propagation, with sensitivity variations exceeding 30%. These findings also demonstrated the limitations of using strain alone to assess ASB formation. It is worth noting, however, that Zhang’s work focused on idealized textures and did not account for the complexities introduced by commercially processed titanium alloys. Therefore, a comprehensive understanding of how initial textures developed under industrial processing routes affect ASB susceptibility is essential for improving material reliability and mitigating dynamic failure in practical applications.

To address these limitations, this study investigates the dynamic compressive behavior and adiabatic shear band sensitivity of extruded and annealed commercial Ti-6Al-4V (TC4) alloy bars subjected to loading along the extrusion direction (ED) and transverse direction (TD). Dynamic compression experiments were conducted using a split Hopkinson pressure bar (SHPB) apparatus, complemented by in situ digital image correlation (DIC) to capture the evolution of strain localization. To complement the experimental results and provide a comprehensive understanding of the deformation behavior at both macroscopic and microscopic scales, a dislocation density-based crystal plasticity finite element model (CPFEM) was employed. This work aims to elucidate how initial texture influences the dynamic mechanical response and ASB sensitivity of TC4 alloys. The results provide guidance for safe design and microstructural optimization of titanium alloys in high-rate engineering applications.

## 2. Materials and Methods

### 2.1. Materials and Heat Treatment

**Figure 1:**
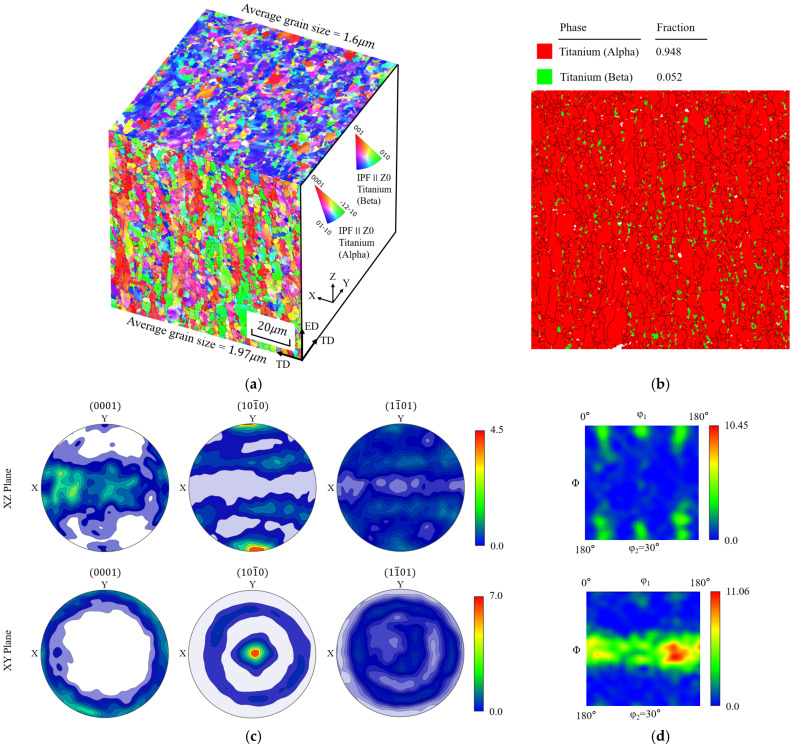
Microstructural characterization of the TC4 alloy: (**a**) inverse pole figures (IPFs) obtained from the XY and XZ planes, together with the corresponding average grain sizes; (**b**) phase distribution and grain boundary map; (**c**) pole figures (PFs) of the (0001), ($10\bar{1}0$), and ($1\bar{1}01$) planes (white regions indicate areas where crystallographic orientations could not be indexed); (**d**) orientation distribution functions (ODFs).

The experimental specimens were prepared from extruded and annealed commercial TC4 alloy rods, whose chemical composition is listed in Table 1. After extrusion, the material was heated to 760–780 °C and furnace-cooled. As shown in Figure 1b, the alloy consists predominantly of approximately 95% α phase (hexagonal close-packed, HCP) and about 5% finely dispersed β phase (body-centered cubic, BCC). Scanning electron microscopy (SEM) was performed on both the axial (extrusion direction, ED) and radial (transverse direction, TD) cross-sections at the center of the bar using a Thermo Scientific Apreo 2C SEM (Thermo Fisher Scientific, Waltham, MA, USA). Electron backscatter diffraction (EBSD) analysis was conducted at an accelerating voltage of 20 kV, and the data were processed using AztecCrystal (2.1.2) and MTEX (5.10.0) software to characterize grain morphology and crystallographic texture.

Figure 1a presents the inverse pole figures (IPFs) for the two cross-sectional orientations. The XY plane (ED plane) exhibits a homogeneous equiaxed grain structure, whereas the XZ plane (TD plane) shows a mixed distribution of equiaxed grains and grains elongated along the axial direction. The average grain size is 1.97 μm on the ED plane and 1.60 μm on the TD plane. Statistical analysis indicates that the weighted standard deviations are 0.98 μm for the ED plane and 1.03 μm for the TD plane, suggesting a comparable degree of grain size dispersion in both loading orientations. Figure 1c displays the pole figures (PFs) of the (0001), $\left(10\bar{1}0\right)$, and $\left(1\bar{1}01\right)$ planes extracted from the two sections. On the XZ plane, the PFs exhibit weak, dispersed strip-like intensity distributions extending along the x-direction, indicating a relatively weak overall texture. In contrast, the ($10\bar{1}0$) PF of the XY plane shows a pronounced central intensity peak with nearly concentric isointensity contours, characteristic of a strong prismatic ⟨a⟩ fiber texture, in which most grain *c*-axes tend to align parallel to the observation plane. The J-index of the XY plane (J = 4.31) is nearly twice that of the XZ plane (J = 2.31), indicating a significantly stronger crystallographic texture and a higher degree of orientation concentration in the XY plane. Figure 1d presents the orientation distribution functions (ODFs). The XZ plane exhibits a pronounced and continuous high-intensity band, indicating strong alignment of grain orientations. In contrast, the XY plane shows a more diffuse texture distribution, reflecting greater orientation heterogeneity.

### 2.2. Specimen Design and Test Procedures

Cubic specimens with dimensions of 4 mm × 4 mm × 4 mm were fabricated for dynamic compression testing. The square cross-section facilitates lateral observation during loading. This geometry corresponds to an equivalent length-to-diameter (L/D) ratio of 1, which is favorable for maintaining mechanical equilibrium and achieving a stable strain-rate plateau during split Hopkinson pressure bar (SHPB) experiments [14]. The stress (σ), strain (ε), and strain rate ($\dot{\varepsilon }$) can be determined using the following equations:

(1) $$\sigma \left(t\right)=\frac{EA}{A_{s}}\varepsilon_{t}\left(t\right)$$

(2) $$\varepsilon \left(t\right)=-2\frac{C_{0}}{L}\int_{0}^{t}\varepsilon_{r}\left(t\right)dt=-2\frac{C_{0}}{L}\int_{0}^{t}\left[\varepsilon_{t}\left(t\right)-\varepsilon_{i}\left(t\right)\right]dt$$

(3) $$\dot{\varepsilon }\left(t\right)=-2\frac{C_{0}}{L}\varepsilon_{r}\left(t\right)=-2\frac{C_{0}}{L}\left[\varepsilon_{t}\left(t\right)-\varepsilon_{i}\left(t\right)\right]$$

where *E* is the elastic modulus of the Hopkinson bar material; *A* is the cross-sectional area of the pressure bars; $A_{s}$ is the cross-sectional area of the specimen; and $\varepsilon_{i}(t)$, $\varepsilon_{t}(t)$ and $\varepsilon_{r}(t)$ denote the incident, transmitted, and reflected strain signals, respectively. L is the length of the pressure bar, and $C_{0}$ is the elastic wave speed in the bar material.

**Figure 2:**
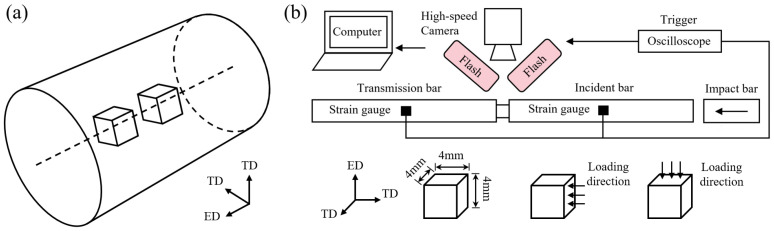
(**a**) Sampling location within the TC4 alloy bar, specimen geometry (4 mm × 4 mm × 4 mm), and loading orientation for SHPB compression tests; (**b**) split Hopkinson pressure bar (SHPB) apparatus.

The specimens were extracted from the central region of TC4 alloy bars with a diameter of 20 mm. Wire electrical discharge machining (EDM) was employed to obtain the initial blanks, followed by precision machining to achieve the designed dimensions and required surface quality. As shown in Figure 2a, the specimens were prepared perpendicular to the rod axis and centered at the midsection to ensure consistent microstructural conditions across all samples.

**Figure 3:**
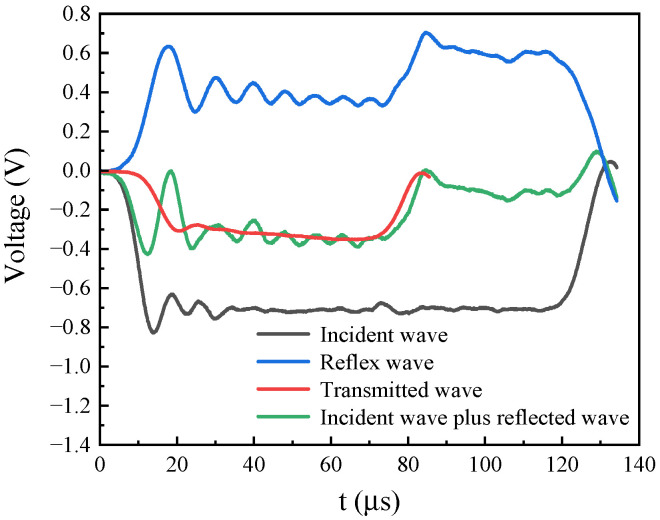
Verification of dynamic stress equilibrium in SHPB compression using the two-wave method: comparison between the superposition of the incident and reflected waves and the transmitted wave.

As shown in Figure 2b, dynamic uniaxial compression tests were conducted using a split Hopkinson pressure bar (SHPB) system. Both the incident and transmission bars were fabricated from 18Ni maraging steel, with identical dimensions of 14.5 mm in diameter and 1000 mm in length. A striker bar with a length of 300 mm was used to impact the specimens along both the extrusion direction (ED) and the transverse direction (TD). Strain signals were recorded using resistance strain gauges mounted on the incident and transmission bars, and the nominal stress–strain responses were calculated based on one-dimensional elastic wave theory. To verify dynamic stress equilibrium during the SHPB tests, a two-wave analysis was performed by directly comparing the superposition of the incident and reflected waves (σ_inc_ + σ_ref_) with the transmitted stress wave (σ_trans_). As shown in Figure 3, the σ_inc_ + σ_ref_ curve closely overlaps with σ_trans throughout the main deformation stage. This agreement confirms that dynamic stress equilibrium was achieved within the specimen, ensuring the validity of the measured dynamic stress–strain responses. To capture the full-field deformation behavior, a Phantom high-speed camera was synchronized with the dynamic loading. Random speckle patterns were generated on the specimen surfaces by laser etching, and the strain-field evolution was analyzed using the digital image correlation (DIC) software MatchID (version 2022.2.3). In the DIC analysis, a subset size of 11 pixels, a step size of 1 pixel, and a strain window of 9 pixels were adopted to ensure sufficient spatial resolution for characterizing strain localization.

## 3. Results

### Stress–Strain Response and Strain Field Evolution Under Dynamic Compression

**Figure 4:**
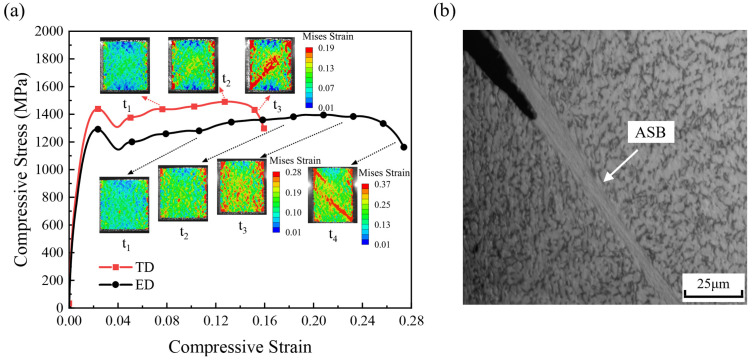
(**a**) Compressive stress–strain responses and corresponding DIC strain-field images of TC4 specimens subjected to dynamic compression along the ED and TD directions at a strain rate of 2500 s^−1^; (**b**) metallographic evidence of adiabatic shear failure in TC4 alloy.

Figure 4a presents the compressive stress–strain responses and the evolution of strain fields during dynamic compression along the ED and TD directions. Each test was repeated 3 times under identical loading conditions, and the resulting stress–strain curves exhibited good repeatability, with stress deviations between repeated tests not exceeding 30 MPa. During dynamic compression along the ED direction, the high-speed camera recorded the TD surface of the specimen. Therefore, all subsequent results are named according to the observation surface. Under the same strain rate, the two loading directions exhibit distinctly different mechanical responses. The TD direction shows a yield strength approximately 100 MPa higher than that of the ED direction, and this difference is significantly larger than the experimental scatter, indicating a pronounced texture dependence even at the yielding stage. After yielding, both directions exhibit similar strain hardening behavior, and their stress–strain curves remain nearly parallel over a wide strain range. At this stage, strain heterogeneity begins to develop within the specimen. This behavior primarily arises from crystallographic orientation differences among grains, which lead to variations in slip system activation sequences and activation resistance. Grains or regions with favorable orientations are activated earlier and exhibit local plastic strain rates higher than the macroscopic average [15], thereby resulting in a non-uniform strain distribution within the material. As deformation proceeds, the TD specimens exhibit stress softening followed by rapid stress collapse at a compressive strain of 0.13. In contrast, the ED specimen continues to harden at a similar rate until softening begins at a compressive strain of 0.23, with a slower stress collapse compared to the TD direction. The strain field evolution captured by the high-speed camera, combined with digital image correlation (DIC), further reveals differences in the adiabatic shear band (ASB) formation process between the two loading directions. At an early stage of plastic deformation (at t_1_), both specimens exhibit non-uniform strain distributions with the emergence of localized strain regions. As the strain increases to approximately 0.13, strain localization becomes more pronounced, but their spatial distribution differs significantly between the two textures. In the ED specimen, localized strain regions remain dispersed and spatially separated, whereas in the TD specimens, they tend to align diagonally. With further deformation, a continuous shear band forms in the TD specimens when the macroscopic compressive strain reaches approximately 0.154, coinciding with the onset of macroscopic stress softening. At the same strain level, although the ED specimen also shows more pronounced strain localization, the bands remain discontinuous. Continuous shear band formation in the ED direction is only observed at a much higher strain of approximately 0.27.

Figure 4b presents the metallographic observations of the axial sections of the recovered specimens. The results indicate that, under dynamic compressive loading, TC4 specimens exhibit adiabatic shear failure in both the ED and TD loading directions. The shear band formed under TD loading makes an angle of approximately 47° with respect to the loading direction, whereas that under ED loading forms an angle of approximately 45°. In both cases, the shear bands display a distinct planar morphology, and no conical fracture surface is observed. Based on multiple repeated experiments, the macroscopic peak stress can be reasonably considered the criterion for the initiation of adiabatic shear bands (ASBs). Compared with the ED direction, ASB initiation and the subsequent failure process in the TD direction occur at an earlier stage, indicating greater sensitivity to adiabatic shear localization.

It is worth noting that the rapid stress drop observed at a macroscopic strain of approximately 0.02 is primarily associated with the transient dynamic response of the split Hopkinson pressure bar (SHPB) system during the initial loading stage—such as wave dispersion, inertia coupling, and transient specimen–bar interactions—rather than with intrinsic thermal softening or damage mechanisms of the material.

## 4. CPFEM Simulations of Dynamic Compression in Fiber Texture TC4 Alloy

Due to the pronounced spatial complexity and heterogeneity of the initial texture in the experimental rod, it is challenging to isolate the specific contributions of individual texture components to ASB formation through experimental approaches alone. In addition, post-mortem, non-in situ observations of failed specimens cannot fully capture the role of texture during the evolution of strain localization. To address these limitations, crystal plasticity simulations were performed using the open-source materials simulation toolkit DAMASK [16], coupled with the ABAQUS finite element solver. A dislocation density-based crystal plasticity constitutive model was implemented to further elucidate the mechanisms by which texture influences sensitivity to adiabatic shear localization. Considering the extremely low volume fraction of the β phase in the experimental TC4 alloy and the fact that plastic deformation anisotropy is predominantly governed by the HCP-structured α phase, a single-phase polycrystalline α model was adopted for the CPFEM simulations.

### 4.1. Constitutive Law

**Figure 5:**
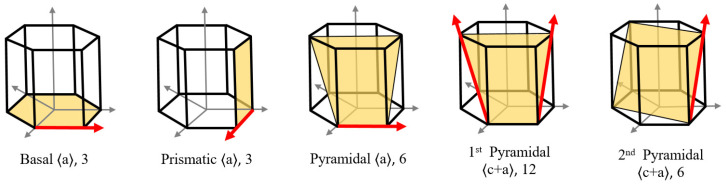
Slip systems in an HCP single crystal. (The arrows indicate the slip directions of the corresponding slip systems).

The dislocation density-based crystal plasticity model follows the approach proposed by Ma and Roters [17] and is implemented within a finite element framework for dislocation-mediated crystal plasticity [18,19]. The shear slip rate on slip system α is described by the dislocation-based activation energy model [20] as

(4) $${\dot{\gamma }}^{\alpha }=\rho^{\alpha }b_{s}\nu_{0}\exp\left[-\frac{Q_{s}}{k_{B}T}{\left\{1-{\left(\frac{\left|\tau_{\mathrm{eff}}\right|}{\tau_{\mathrm{sol}}}\right)}^{p}\right\}}^{q}\right]\mathrm{sgn}(\tau^{\alpha })$$

where $\rho^{\alpha }$ is the dislocation density on the slip system α, $b_{s}$ is the Burgers vector, $v_{0}$ is the reference shear velocity, $Q_{s}$ is the total short-range obstacle energy (i.e., the slip system activation energy), $k_{B}$ is the Boltzmann constant, T is the absolute temperature, and $\tau_{\mathrm{sol}}$ is the solute strength, whose magnitude depends on both the strain rate and the alloying elements [21,22]. The parameters 0 ˂ p ≤ 1 and 1 ≤ q≤2 [20] govern the shape of the short-range energy barrier. The thermally activated component of the effective shear stress $\tau_{eff}$ is defined as

(5) $$\tau_{\mathrm{eff}}=\left\{\begin{array}{cc} \left|\tau^{\alpha }\right|-\tau_{\mathrm{pass}}^{\alpha } & \mathrm{for}\left|\tau \right|>\tau_{\mathrm{pass}}\\ 0 & \mathrm{for}\left|\tau \right|\leq \tau_{\mathrm{pass}} \end{array}\right.$$

where $\tau^{\alpha }$ is the total shear stress component on the slip system α, while $\tau_{pass}^{\alpha }$ is the threshold stress for overcoming long-range obstacles, defined as follows:

(6) $$\tau_{\mathrm{pass}}^{\alpha }=Gb_{s}^{\alpha }{\left(\sum_{\alpha^{\prime }=1}^{N_{s}}h^{\alpha \alpha^{\prime }}(\rho^{\alpha^{\prime }}+\rho_{\mathrm{di}}^{\alpha^{\prime }})\right)}^{1/2}$$

where G is the shear modulus; $\rho^{\alpha^{\prime }}$ and $\rho_{di}^{\alpha^{\prime }}$ are the dislocation density and dislocation dipole density, respectively, on the $\alpha^{\prime }$ slip system; and $h^{\alpha \alpha^{\prime }}$ is the interaction matrix between slip systems α and $\alpha^{\prime }$, determined via discrete dislocation dynamics (DDD) simulations [23]. The slip systems in the hexagonal close-packed (HCP) crystal structure are shown in Figure 5.

The evolution of dislocation density is governed by the competing mechanisms of dislocation multiplication and annihilation, and their respective evolution equations are given as follows [24,25]:

(7) $${\dot{\rho }}^{\alpha }=\frac{\left|{\dot{\gamma }}^{\alpha }\right|}{b_{s}\Lambda_{s}}-\frac{2\overset{\wedge }{d}}{b_{s}}\rho |{\dot{\gamma }}^{\alpha }|$$

(8) $${\dot{\rho }}_{\mathrm{di}}^{\alpha }=\left\{\begin{array}{cc} \frac{2(\overset{\wedge }{d}-\overset{\vee }{d})}{b_{s}}\rho^{\alpha }|{\dot{\gamma }}^{\alpha }|-\frac{2\overset{\vee }{d}}{b_{s}}\rho_{\mathrm{di}}^{\alpha }|{\dot{\gamma }}^{\alpha }|-\rho_{\mathrm{di}}^{\alpha }\frac{4\nu_{\mathrm{cl}}}{\overset{\wedge }{d}-\overset{\vee }{d}} & \mathrm{for}\;\hat{d}\leq \Lambda_{s}\\ 0 & \mathrm{for}\;\hat{d}>\Lambda_{s} \end{array}\right.$$

The first term in Equation (4) describes the accumulation of dislocations due to dislocation multiplication, which contributes to strain hardening. This process is characterized by the mean free path of dislocation slip. As no deformation twinning was observed in the experiments, its contribution is neglected in the present analysis. $\Lambda_{s}^{\alpha }$ is defined as follows:

(9) $$\frac{1}{\Lambda_{s}^{\alpha }}=\frac{1}{D}+\frac{1}{\lambda_{s}}$$

where *D* is the average grain size, and

(10) $$\frac{1}{\lambda_{s}^{\alpha }}=\frac{1}{i_{s}}{\left(\sum_{\alpha^{\prime }=1}^{N_{s}}p^{\alpha \alpha^{\prime }}(\rho^{\alpha^{\prime }}+\rho_{\mathrm{di}}^{\alpha^{\prime }})\right)}^{1/2}$$

In this equation, $i_{s}$ is a fitting parameter, and $p^{\alpha \alpha^{\prime }}$ is the projection coefficient of the forest dislocation density on the slip system $\alpha^{\prime }$ onto slip system α.

The second term in Equation (4) captures the decrease in mobile dislocation density due to the formation of dislocation dipoles. In Equation (5), the first term describes dipole formation, while the latter two terms correspond to spontaneous annihilation and climb-induced annihilation of dipoles, respectively. The critical distance $\hat{d}$ for the stable formation of dipoles during dislocation glide is defined as

(11) $$\overset{\wedge }{d}=\frac{3Gb_{s}}{16\pi |\tau |}$$

The critical distance for spontaneous annihilation of dislocation dipoles and the thermally activated climb velocity are given by

(12) $$\overset{\vee }{d}=D_{a}b_{s}$$

(13) $$\nu_{\mathrm{cl}}=\frac{GD_{0}V_{\mathrm{cl}}}{\pi (1-\nu )k_{B}T}\frac{1}{\overset{\wedge }{d}+\overset{\vee }{d}}\exp(-\frac{Q_{\mathrm{cl}}}{k_{B}T})$$

where $D_{a}$ is the dipole annihilation coefficient, $D_{0}$ is the pre-exponential factor of the self-diffusion coefficient, $V_{cl}$ is the activation volume for dislocation climb, $Q_{cl}$ is the activation energy for climb, and v is Poisson’s ratio.

### 4.2. Parameters

The selection of material parameters in crystal plasticity models is critical for accurately predicting material-specific deformation behavior. To reproduce the experimentally observed response, the parameters within the CPFEM constitutive framework must be systematically determined. Although the model involves complex evolution equations, most parameters have clear physical meanings and can be obtained from experimental measurements or reliable literature sources. The remaining adjustable parameters are calibrated through iterative fitting to the experimentally measured stress–strain curves.

The fourth-order elastic tensor of titanium and its alloys has been extensively characterized using various experimental techniques. Heldmann et al. [26] determined the elastic tensor of polycrystalline Ti-6Al-4V via neutron diffraction and provided a comparative overview of relevant measurements spanning several decades. Alternatively, Shi et al. [27] extracted the elastic constants of Ti-6Al-4V based on a resonance frequency method. In the present study, experimentally reported elastic constants from Bacon et al. [28] and Huntington [29] were adopted as input parameters, specifically: C_11_ = 164.2 GPa, C_12_ = 92.0 GPa, C_13_ = 69.0 GPa, C_33_ = 180.7 GPa, and C_44_ = 46.7 GPa. Based on the optimized parameter ranges reported by Tang et al. [30] and Sedighiani et al. [31], the relevant parameters in Equation (1) were selected as follows: the reference dislocation glide velocity $v_{0}=$ 4 × 10^3^ m/s, the activation energy for slip $Q_{s}=$1.8 × 10^−19^ J and the Burgers vector magnitude bs was set to 2.95 × 10^−10^ m for basal and prismatic slip systems and 5.53 × 10^−10^ m for pyramidal ⟨c + a⟩ slip systems. Since $\tau_{sol}$ increases with $v_{0}$ [32], this study used $v_{0}$ = 4 × 10^3^ m/s, corresponding to $\tau_{sol}$ = 600 MPa. In Equation (10), the self-diffusion prefactor $D_{0}$ = 1.38 × 10^−3^ m^2^/s and the climb activation energy $Q_{cl}$ = 1.2 eV were adopted from the results of Köppers et al. [33] and Kannan and Thomas [34], respectively. The remaining evolution parameters were identified through iterative fitting against experimentally obtained macroscopic stress–strain curves: p= 0.72, q= 1.04, $i_{s}=$ 13 and $D_{a}=$ 9.0. The relevant material parameters of the Ti-6Al-4V alloy are summarized in Table 2.

### 4.3. Polycrystalline Crystal Plasticity Model and Shear Boundary Conditions

**Figure 6:**
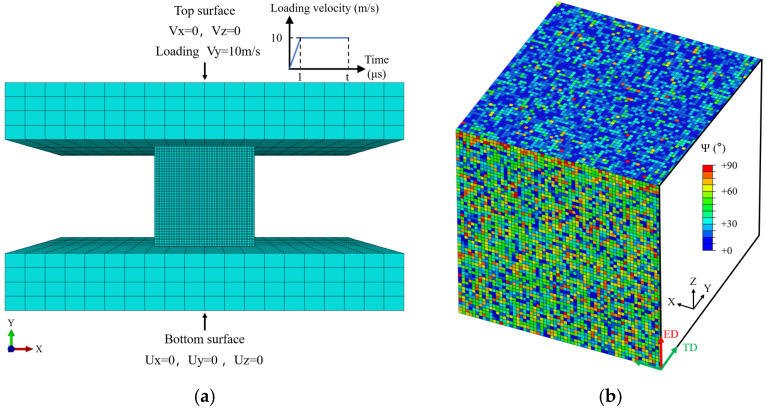
CPFEM model constructed based on the microstructural characteristics of the TC4 alloy bar: (**a**) applied boundary conditions and loading profile used in the simulation; (**b**) grain orientation angles between the *c*-axis and the respective planes for ED- and TD-textured models, obtained from EBSD measurements.

As illustrated in Figure 6a, surface-to-surface contact was defined between the specimen and the loading plates, with a friction coefficient of 0.3 [35]. The simulations indicate that moderate variations in this coefficient exert a limited influence on the strain corresponding to ASB initiation. To simplify the actual microstructure, each finite element was assumed to represent a single grain. The model dimensions are identical to those of the experimental specimen (4 × 4 × 4 mm^3^) and comprise 210,000 C3D8R elements. A peak loading velocity of $V_{\mathrm{peak}}=10$ m/s was applied to achieve the experimental strain rate, consistent with the experimental conditions. Figure 6b shows the angles between the grain *c*-axes and their respective planes in the ED and TD orientation models. Grain orientations obtained from EBSD measurements were spatially mapped onto the corresponding grains in the numerical model, ensuring close consistency between the crystal plasticity simulations and the experimental specimen in terms of crystallographic orientation and texture distribution. The simulated grain orientations closely reproduce those observed on the specimen surface, thereby enhancing the reliability of the model in capturing texture-related deformation behavior. It should be noted that the colored elements in the schematic represent groups of grains with similar orientations; individual orientations are not shown exhaustively. Comparison of the two textures indicates that the ED model exhibits a more dispersed orientation distribution, with grain *c*-axes forming relatively large angles with the XY plane. In contrast, the TD model displays a more concentrated texture, with grain *c*-axes predominantly aligned parallel to the XY plane. This pronounced difference in crystallographic texture forms the basis for analyzing the direction-dependent evolution of strain localization in the subsequent simulations.

### 4.4. CPFEM Simulation Results

**Figure 7:**
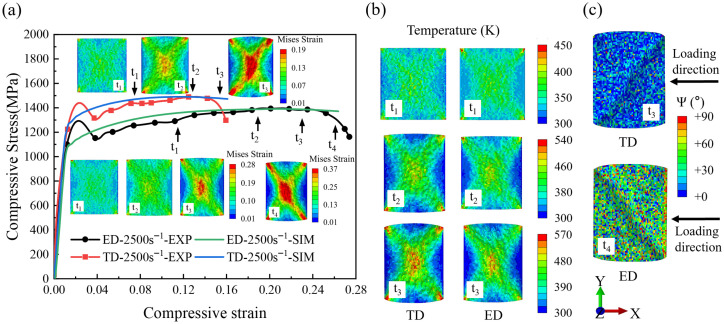
CPFEM simulation results: (**a**) CPFEM-simulated compressive stress–strain responses for TC4 specimens loaded along the ED and TD directions at a strain rate of experiment and strain field distribution at different deformation stages; (**b**) temperature field distribution at different deformation stages; (**c**) spatial distribution of grain *c*-axis orientations.

Figure 7a presents the compressive stress–strain curves predicted by the CPFEM simulations for the ED- and TD-textured models, together with the corresponding deformation fields at different stages of plastic deformation. Upon entering the plastic regime, both models exhibit a brief stress drop following an initial rise. This phenomenon reflects the inherent heterogeneity of plastic deformation in polycrystalline materials, where slip initiates preferentially in favorably oriented grains [15], whose local deformation and strain rate significantly exceed the average. The rapid development of localized plasticity can lead to a reduction in load-bearing capacity, resulting in a stress drop. As deformation proceeds, both texture models exhibit similar strain-hardening behavior. During the strain-hardening stage (at t_1_), strain heterogeneity begins to appear; however, the overall deformation remains approximately homogeneous under compression. Clear differences emerge during dynamic instability. At a compressive strain of approximately 0.13, the TD model exhibits early stress softening accompanied by the formation of a distinct strain localization band. In contrast, the ED model maintains stable hardening to higher strains and does not show stress softening until a strain of approximately 0.23. Moreover, at the onset of localization, strain concentration in the ED direction is weaker and more spatially dispersed. Under identical strain-rate and global strain conditions, the TD model therefore exhibits stronger localization and more rapid instability development. Figure 7b shows the evolution of the temperature field at different plastic stages, which follows trends consistent with the strain-field evolution. Figure 7c presents the distribution of angles between the grain *c*-axis and the respective planes at the final stage of deformation. In the TD model, grains within the shear band display similar *c*-axis orientations, indicating orientation-assisted localization.

Comparison with the experimental results (Figure 4) indicates that the CPFEM-predicted stress–strain responses and strain-field evolution are generally consistent with the observations. The simulations capture the differences in dynamic compressive behavior arising from distinct initial texture conditions, and the predicted localization paths exhibit a planar shear morphology in agreement with the experimental results. Some discrepancies remain in the post-localization stage. In dynamic compression experiments, perfectly symmetric loading conditions are difficult to achieve. Once localization preferentially develops on one side of the specimen, the competing localization path is suppressed, resulting in a single dominant adiabatic shear band. In contrast, the CPFEM simulations are conducted under ideal symmetric boundary conditions, leading to symmetrically distributed shear localization patterns.

Overall, the numerical model effectively reflects the macroscopic mechanical response associated with localization initiation under different initial texture conditions, as well as the corresponding microstructural evolution. It therefore provides a reasonable basis for interpreting the differences in ASB sensitivity between the two loading directions. It should be noted that, since the current crystal plasticity model does not incorporate damage evolution, the simulated stress–strain curves do not exhibit the abrupt stress collapse observed experimentally.

## 5. Discussion

### 5.1. Effect of Texture-Controlled Slip Modes on Flow Stress

Analysis of the crystallographic orientations in the ED and TD specimens reveals pronounced differences in the alignment of the α-phase *c*-axis relative to the loading direction, leading to the activation of distinct slip systems during deformation. For α-titanium, it is well established that prismatic ⟨a⟩ slip systems are the most readily activated, followed by basal ⟨a⟩ slip, whereas pyramidal ⟨c + a⟩ slip systems require significantly higher stresses for activation [36,37]. Using in situ far-field high-energy diffraction microscopy, Wang et al. [38] demonstrated that prismatic ⟨a⟩ slip exhibits the lowest critical resolved shear stress (CRSS), followed by basal ⟨a⟩ slip, while pyramidal ⟨c + a⟩ slip possesses the highest CRSS and is only weakly activated under typical loading conditions. In general, although ⟨c + a⟩ slip systems are difficult to activate due to their high CRSS, they become necessary when deformation occurs parallel to the *c*-axis, as ⟨a⟩-type slip alone cannot satisfy strain compatibility requirements [39]. Yang et al. [40] fabricated TC4 samples with distinct orientation characteristics using selective electron beam melting (SEBM) and showed that, under impact loading, when the α-grain *c*-axis is aligned with the loading direction, deformation is dominated by pyramidal ⟨c + a⟩ slip. This leads to elevated flow stress and a faster temperature rise, thereby enhancing thermal softening and increasing ASB sensitivity. Conversely, when the *c*-axis is perpendicular to the loading direction, prismatic and basal ⟨a⟩ slip systems are more readily activated, resulting in lower ASB sensitivity.

**Figure 8:**
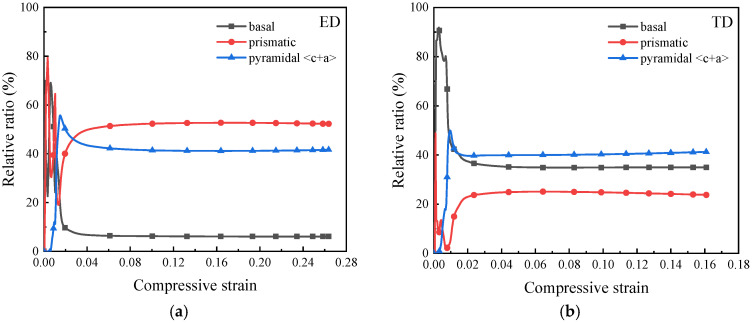

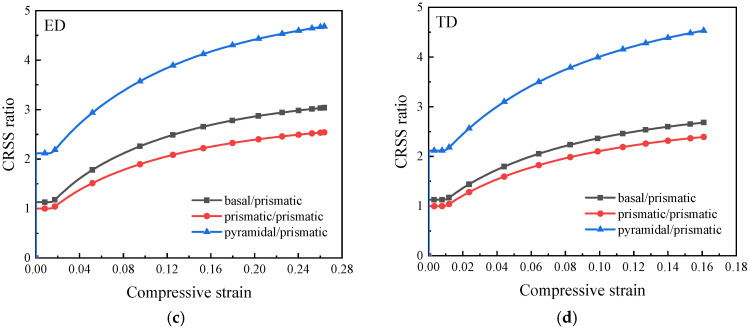
Evolution of slip activity and CRSS during deformation in the ED and TD models: (**a**) relative contribution of each slip system to the total plastic strain in the ED model; (**b**) relative contribution of each slip system to the total plastic strain in the TD model; (**c**) evolution of the critical resolved shear stress (CRSS) of each slip system with increasing strain in the ED model; (**d**) evolution of the CRSS of each slip system with increasing strain in the TD model.

The CPFEM results provide quantitative support for this interpretation. Figure 8 presents the contributions of each slip system to plastic strain and the evolution of CRSS in the ED and TD models. Slip activity was quantified by calculating the fraction of cumulative slip accommodated by each slip system relative to the total slip. The results are shown in Figure 8a,b. For both orientations, the relative contributions approach saturation at a macroscopic strain of approximately 0.025. In the ED model, where grain orientations are more dispersed, prismatic and basal ⟨a⟩ slip systems are activated first due to their lower CRSS. As deformation proceeds, pyramidal ⟨c + a⟩ slip is progressively activated to accommodate strain along the *c*-axis and grain rotation. The final plastic strain contributions are 52% for prismatic ⟨a⟩ slip and 41% for pyramidal ⟨c + a⟩ slip, indicating that deformation is primarily governed by prismatic ⟨a⟩ slip. In the TD model, most grain *c*-axes are aligned with the loading direction, making pyramidal ⟨c + a⟩ and basal ⟨a⟩ slip systems more favorably oriented. Their respective contributions to plastic strain are 41% and 35%, indicating that deformation is primarily controlled by pyramidal ⟨c + a⟩ slip. Figure 8c,d show the evolution of CRSS ratios for each slip system, normalized by the initial CRSS of prismatic ⟨a⟩ slip (90 MPa). At the onset of deformation, the CRSS ratios for basal ⟨a⟩:prismatic ⟨a⟩:pyramidal ⟨c + a⟩ are 1.2:1:2, consistent with experimental measurements reported in the literature [38]. With increasing strain, all slip systems exhibit progressive hardening.

These results demonstrate that the anisotropic flow-stress response under dynamic compression is primarily governed by texture-controlled slip activation. In the ED direction, where deformation is dominated by prismatic ⟨a⟩ slip, the lower CRSS results in a lower macroscopic flow stress. In contrast, in the TD direction, pyramidal ⟨c + a⟩ slip contributes more significantly to plastic deformation, and its higher CRSS leads to an elevated overall flow stress. This texture-induced difference in slip behavior establishes the mechanical foundation for the subsequent divergence in strain localization and adiabatic shear band sensitivity discussed in the next section.

### 5.2. Evolution of Deformation Localization

Under dynamic compression at a strain rate of 2500 s^−1^, deformation occurs over a sufficiently short time scale that heat exchange with the surroundings can be neglected. The process can therefore be approximated as adiabatic. In the CPFEM simulations, the adiabatic temperature rise is expressed as

(14) $$\dot{T}=\frac{\beta }{\rho C_{P}}\sigma :{\dot{\varepsilon }}^{p}$$

where $\sigma :{\dot{\varepsilon }}^{p}$ is the plastic work rate per unit volume, ρ is the material density, $C_{P}$ is the specific heat capacity, and β is the fraction of plastic work converted into heat, commonly known as the Taylor–Quinney coefficient [41]. The material parameters used in this study were β = 0.9, ρ = 4430 kg/m^3^, $C_{P}$ = 577 J/(kg·K) [42].

**Figure 9:**
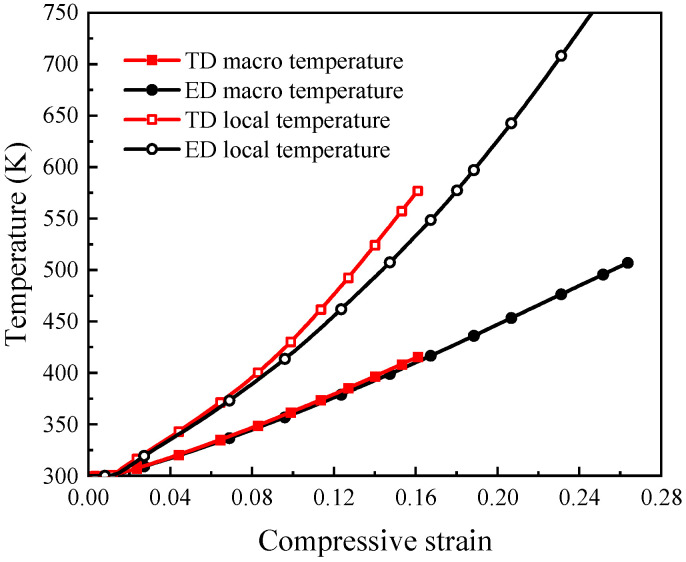
Temperature evolution during dynamic deformation: Evolution of the macroscopic temperature and the local temperature within the shear band region for the ED and TD directions.

Figure 9 compares the evolution of the macroscopic average temperature and the local temperature within the shear band for specimens loaded along the ED and TD directions. Due to the experimental limitations associated with in situ temperature measurement at high strain rates, the temperature results are intended to reveal relative trends rather than absolute values. The TD specimens exhibit a higher macroscopic temperature rise than the ED specimens at the same global strain. This behavior arises from the higher flow stress in the TD direction, which results in greater plastic work accumulation. When adiabatic shear localization initiates in the TD direction at a compressive strain of approximately 0.13, the corresponding macroscopic temperature rise is about 88 K, approximately 6 K higher than that in the ED direction at the same strain. In contrast, ASB formation in the ED direction occurs at a higher compressive strain of approximately 0.23, where the macroscopic temperature rise reaches about 173 K. Although the difference in bulk temperature rise between the two orientations is relatively modest, a more pronounced distinction is observed in the localized temperature within the shear band. At the onset of ASB formation, the local temperature rise in the TD direction reaches approximately 277 K, about 54 K higher than that in the ED direction. The substantially higher local temperature rise in the TD specimens intensifies thermal softening and amplifies initial perturbations in plastic flow, thereby accelerating deformation instability and promoting earlier ASB initiation [43,44]. These results indicate that localized thermal concentration plays a critical role in adiabatic shear band initiation under adiabatic conditions.

In addition to thermal effects, crystallographic texture plays a critical role in the evolution of deformation localization. Both experimental observations and crystal plasticity simulations indicate that slip transfer is more readily facilitated between grains with similar orientations, enabling long-range connectivity of localized deformation paths and promoting ASB development [15]. Dislocation-based CPFEM simulations by Zhang et al. [13] further demonstrated that ASBs tend to initiate and propagate along bands of similarly oriented grains and that the texture significantly affects the onset strain and evolution pattern of ASBs. In the present study, the TD direction exhibits a more concentrated grain orientation distribution, which enhances geometric compatibility across grain boundaries and facilitates intergranular slip transfer. This microstructural configuration accelerates the establishment of continuous strain-localization paths and promotes the rapid formation of adiabatic shear bands, as reflected by the high orientation consistency within localized regions shown in Figure 7c. As a result, the TD direction demonstrates greater sensitivity to ASB formation. In contrast, the ED direction features a more dispersed orientation distribution, leading to spatially isolated strain-localization regions during the early stages of deformation. Continuous shear bands are less likely to form at low strains and instead develop through the progressive coalescence of individual localized zones at higher overall strains, corresponding to reduced susceptibility to adiabatic shear banding.

## 6. Conclusions

In this study, the dynamic compressive behavior and adiabatic shear band (ASB) sensitivity of extruded and annealed Ti-6Al-4V alloy bars were systematically investigated by combining split Hopkinson pressure bar (SHPB) experiments with dislocation density-based crystal plasticity finite element modeling (CPFEM). The results elucidate how texture-controlled anisotropy in the α-phase governs the mechanical response and slip system activation under directional loading, thereby leading to distinct shear localization tendencies and pronounced differences in ASB susceptibility. The main conclusions are as follows:

1. In extrusion-annealed TC4 alloy rods, the α phase exhibits pronounced texture heterogeneity across different sections. The axial (ED) plane shows a relatively uniform texture, whereas the radial (TD) plane develops a typical prismatic ⟨a⟩ fiber texture.
2. Under dynamic compression at 2500 s^−1^, the TD direction exhibits a yield strength approximately 100 MPa higher than that of the ED direction. This anisotropy arises from texture-controlled differences in slip system activation. Specifically, when the *c*-axis is nearly parallel to the loading direction in the TD orientation, plastic deformation is predominantly accommodated by pyramidal ⟨c + a⟩ slip. Due to its higher critical resolved shear stress (CRSS), this slip mode leads to an elevated macroscopic flow stress.
3. For the TC4 alloy investigated herein, adiabatic shear localization in both loading directions initiates near the peak stress. The initial texture significantly influences ASB sensitivity: ASB initiation occurs at a compressive strain of 0.13 in the TD direction, markedly earlier than in the ED direction (0.23), indicating greater ASB sensitivity in the TD orientation.
4. The enhanced ASB sensitivity in the TD orientation originates from the synergistic interaction between thermomechanical coupling and texture-assisted strain localization. On the one hand, the higher flow stress promotes a more rapid temperature rise and intensified thermal softening, thereby accelerating deformation instability. On the other hand, the concentrated grain orientation distribution facilitates intergranular slip transfer, promoting the formation of continuous shear bands.
5. From an engineering perspective, tailoring the microtexture to promote prismatic ⟨a⟩ slip while encouraging spatially dispersed crystallographic orientations represents an effective strategy for mitigating ASB sensitivity in Ti-6Al-4V under high strain-rate loading.

## Figures and Tables

**Table 1:** Chemical composition of the Ti-6Al-4V investigated (wt%).

Element	Ti	Al	V	Fe
wt (%)	89.1	6.3	4.3	0.3

**Table 2:** Constitutive parameters of the material.

Parameter	Basal ⟨a⟩	Prismatic ⟨a⟩	Pyramidal ⟨c + a⟩	Origin
$C_{11}(Gpa)$	162.4			[28,29]
$C_{12}(Gpa)$	92.0			[28,29]
$C_{13}(Gpa)$	69.0			[28,29]
$C_{33}(Gpa)$	180.7			[28,29]
$C_{44}(Gpa)$	46.7			[28,29]
$Q_{s}(J)$	1.8 × 10^−19^	1.8 × 10^−19^	1.8 × 10^−19^	[30]
$\rho_{0}(m/m^{3})$	1.2 × 10^13^	1.2 × 10^13^	1.2 × 10^13^	(This Work)
$b_{s}(m)$	2.95 × 10^−10^	2.95 × 10^−10^	5.53 × 10^−10^	[30]
p	0.72	0.72	0.72	(Fitted)
q	1.04	1.04	1.04	(Fitted)
$v_{0}(m/s)$	4.0 × 10^3^	4.0 × 10^3^	4.0 × 10^3^	(This Work)
$\tau_{sol}(Pa)$	6.0 × 10^8^			(This Work)
$k_{B}(JK^{-1})$	1.38 × 10^−23^			-

## Data Availability

The original contributions presented in this study are included in the article. Further inquiries can be directed to the corresponding author.
